# Epigenetic Regulation of Bone Marrow Stem Cell Aging: Revealing Epigenetic Signatures associated with Hematopoietic and Mesenchymal Stem Cell Aging

**DOI:** 10.14336/AD.2017.1213

**Published:** 2019-02-01

**Authors:** Dimitrios Cakouros, Stan Gronthos

**Affiliations:** ^1^Mesenchymal Stem Cell Laboratory, Adelaide Medical School, Faculty of Health and Medical Sciences, University of Adelaide, Adelaide, SA, Australia.; ^2^South Australian Health and Medical Research Institute, Adelaide, SA, Australia.

**Keywords:** histone, chromatin, HSC, MSC, methylation

## Abstract

In this review we explore the importance of epigenetics as a contributing factor for aging adult stem cells. We summarize the latest findings of epigenetic factors deregulated as adult stem cells age and the consequence on stem cell self-renewal and differentiation, with a focus on adult stem cells in the bone marrow. With the latest whole genome bisulphite sequencing and chromatin immunoprecipitations we are able to decipher an emerging pattern common for adult stem cells in the bone marrow niche and how this might correlate to epigenetic enzymes deregulated during aging. We begin by briefly discussing the initial observations in yeast, drosophila and Caenorhabditis elegans (*C. elegans*) that led to the breakthrough research that identified the role of epigenetic changes associated with lifespan and aging. We then focus on adult stem cells, specifically in the bone marrow, which lends strong support for the deregulation of DNA methyltransferases, histone deacetylases, acetylates, methyltransferases and demethylases in aging stem cells, and how their corresponding epigenetic modifications influence gene expression and the aging phenotype. Given the reversible nature of epigenetic modifications we envisage “epi” targeted therapy as a means to reprogram aged stem cells into their younger counterparts.

## Stem Cell Chromatin Structure

Genome wide maps of epigenetic modifications from mouse and human ESC reveal wide spread active chromatin marks consisting of both histone acetylation and H3K4me3 together with hypomethylated DNA [1]. As these marks contribute to the open chromatin configuration, this ensures the stem cell associated genes are active and that developmental genes contain an open chromatin configuration to maintain an activated status. In order to maintain pluripotency, however, lineage genes must be repressed to ensure stem cells remain immature yet amenable to activation, hence the presence of the bivalent mark consisting of both the repressive H3K27me3 and the active H3K4me3 [1-3]. This is also evident in some adult stem cells such as HSC [4]. During differentiation of ESC there is a transition from the open chromatin structure to a more compact and less permissive structure [5, 6]. This ensures the differentiated cells progress down the lineage they are destined and therefore cannot differentiate into other cell types and is closely associated with the bivalent histone mark resolving to become univalent. Genome wide analysis of H3K9me2 has identified significant increases and chromosome spreading during the process of differentiation [7, 8]. The same is evident for H3K27me3, hence reducing plasticity and enforcing commitment to the desired lineage [9]. Due to the scarcity of obtaining pure tissue specific stem cells only a few studies have been conducted examining the genome wide distribution of epigenetic changes. Reports so far show that adult stem cells represent an intermediate between pluripotent and terminally differentiated stem cells. A common set of stem associated genes including chromatin regulators, transcription, cell cycle and survival genes are marked with H3K4me3 in multiple adult stem cells [10].

## The Epigenetic link with Aging

The connection between chromatin and the aging process first came to our attention more than two decades ago using the budding yeast, *S. cereviasae*. This unicellular organism has been invaluable in biochemical and genetic studies owing to the ease of performing complex genetic manipulations in an era when performing similar experiments in human cells was not feasible. Being a unicellular organism, *S. cerevisae* is an ideal model to investigate the mechanisms of aging as measured by assaying the replicative lifespan or the number of daughter cells each mother cell can produce before entering senescence. Initial studies reported that aging in yeast correlated with a loss of heterochromatin silencing at telomeres, the mating type locus and ribosomal DNA repeats [11, 12]. Direct involvement of histones in the process of aging is illustrated when yeast cells deficient in the histone chaperone, Asf1, displayed lower histone levels correlating to a shorter lifespan [13, 14], in agreement with the observation that histone levels themselves decline with age [15]. When histone levels are raised, the life span is increased considerably [13]. These results imply that the failure to maintain proper chromatin structure is a pivotal causative factor of the aging process.

In mammalian cells, the irreversible block in proliferation otherwise known as senescence is a contributing factor to the aging process. This process is well characterized by the presence of dense non-pericentromeric heterochromatin termed senescence associated heterochromatin foci, which have high levels of H3K9me3 and H3K27me3 [16-19]. Genome wide studies involving ChiPseq analyses mapped H3K27me3 and H3K9me3 to large contiguous regions corresponding to lamin associated domains (LAD) [20]. Senescence associated changes in these histone marks also correlated with senescence associated gene expression changes with loss of H3K4me3 at down-regulated genes and loss of H3K27me3 at up-regulated genes [21]. A screen to identify heterochromatic gene silencing identified Sir2 in yeast, which was associated with longevity [22]. Sir2 is an NAD+ dependent histone deacetylase and part of the sirtuin family, and its discovery supports the heterochromatin loss model of aging where the disregulation of heterochromatin in a cell increases with aging [23-26]. Sir2 normally deacetylates H4K16 and in yeast cells Sir2 levels normally decrease with age, which corresponds to an increase in H4K16 acetylation [27]. Genome wide aging studies in Drosophila, reported a general decrease in active chromatin marks H3K4me3 and H3K36me3. The most significant change however was the decrease in the enrichment of the repressive heterochromatin mark H3K9me3 and its associated protein, heterochromatin protein 1 (HP1) at pericentric heterochromatin. Genes that lost these marks showed an increase in transcription with age [28]. To elucidate the function of HP1/heterochromatin in aging, knocking out HP1 in flies resulted in reduced lifespan, whereas overexpressing HP1 resulted in increased lifespan [29]. The loss of heterochromatin regions is now an established phenomenon associated with aging. However, phenotypic effects associated with histone marks and aging seem to be specific to each mark. This is evident with H3K27me3, which is associated with repression and genetic mutations in the H3K27 methyltransferase in drosophila resulting in an increase in life span [30]. These findings highlight that histone marks are located on specific regions of the genome affecting specific functions and that there also could be tissue specific differences.

The association between histone methylation and lifespan was demonstrated using a targeted siRNA screen in *C. elegans*. Deletion of the H3K4 methyltransferases, Ash2, set-2 and set-4 (which have human homologous) extended the worms lifespan [31] with Ash2 having the greatest effect. In agreement with these findings, the elimination of the H3K4 demethylase, RBR2, homologous to human RBP2 and PLU1, resulted in a reduced lifespan. Knockdown of the worm orthologue, LSD1, a H3K4 and H3K9 demethylase leads to an increase in lifespan [32]. These studies illustrate a paradox that different enzymes affecting the same histone modification have different outcomes. This is likely to different genes being targeted by the different enzymes which could also be different in different organisms. Elegant studies examining the function of H3K27me3 in worms has found that the deletion of the H3K27 demethylase, UTX results in increased levels of H3K27 in the genome and increased lifespan [33, 34] The key genes affected in this study revealed that the insulin signalling pathway was a critical driver for the life span phenotype and suggested that modification of the aging induced epigenetic signatures to a more naïve immature epigenetic status might be essential in delaying the aging process [33, 34].

The first ever histone modifying protein to be discovered to have a link with aging is the Sirtuin protein. Sirtuins are class III NAD+ dependent histone deacetylases [22] [35, 36]. There are seven mammalian sirtuins with Sirt1 being the closest homolog of *s. cereviasiae* Sir2 [37]. Sir2 is essential in maintaining the heterochromatin structure in regions adjacent to telomeres, at the silent mating type loci and at ribosomal DNA repeats [38]. In mice, loss of Sirt1 results in heart and retinal abnormalities, defective gametogenesis, genomic instability and reduced survival [39-41]. Sirt1 targets expand further than histone proteins, affecting stress responses, mitochondrial biogenesis, adipogenesis, osteogenesis, glycogenesis, genomic integrity and the inflammatory responses [42]. During aging, the levels of Sirt1 decline contributing to most of the aging phenotypes [43]. Another mammalian member, Sirt6 specifically deacetylates H3K9 and H3K56 [44, 45]. Sirt6 associates with telomeres promoting a repressive heterochromatin structure, and is important for maintaining genomic integrity [42], where removal of Sirt6 accelerates aging. Further support for histone deacetylation in aging comes from the use of HDAC inhibitors, which can delay age dependent neurodegeneration and progression of Alzheimer’s Disease in animal models leading to an increase in learning ability ([46, 47]. Furthermore, HDAC inhibitors have been shown to increase lifespan in worms [48]. Once again, there is a disparity showing that different histone deacetylases have different effects on longevity depending on gene targets, tissue and organism.

**Figure 1.**
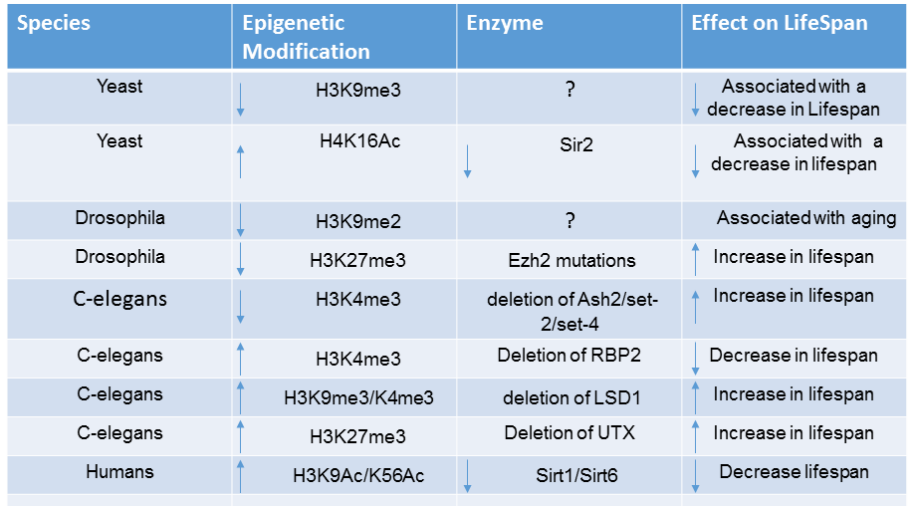
**Age Associated Epigenetic Changes in Organisms**. Histone and DNA modification marks associated with aging in different species is shown as well as their associated epigenetic enzymes. The effect on aging and lifespan is also shown.

Diseases associated with premature aging have been vital in identifying genes deregulated in this process. The role of chromatin modifications and remodelling is underscored in Hutchinson Gilford Progeria Syndrome as there is a decrease in H3K9me3, increase in H4K20me3 [49], and increase in DNA damage accumulation partly due to the decrease in histone associated proteins RBBP4 and RBBP7 [50]. In mouse models of Progeria, supplementing mice with HDAC inhibitors resulted in decreased senescence, more efficient DNA damage repair and extended lifespan [51, 52]. A table outlining the role of epigenetic modifiers and modifications in longevity in organisms can be seen in Figure 1.

## Epigenetics in Stem Cell Aging

Genome wide studies examining epigenetic regulation in terminally differentiated somatic cells have been informative in understanding how histone or DNA marks are distributed in different regions of the genome. For example, epigenetic marks can be preferentially present along enhancers, transcription start sites, exon/intron boundaries, untranslated regions and centromeres. The preferential location of different marks gives us an insight into their function in regulating gene expression. In more recent times, different stem cell populations have been examined on a genome wide basis to understand how these epigenetic marks are involved in governing self-renewal or lineage commitment processes. This has been made possible by advances in methodologies that identify and purify stem cell populations, however obtaining sufficient yields of chromatin and/or DNA derived from rare stem cell populations has hampered genome wide ChIPseq and DNAseq studies. To date, very few studies have examined epigenetic modifications in adult stem cells of young and aged donors to examine how changes in epigenetic signatures are associated with a decline in stem cell number and function during aging. This notion is primarily based on investigations into the role of epigenetics in hematopoietic stem cell maintenance and function during aging.

The epigenetic determinants as causative factors associated with aging is a fascinating field that promises the use of epigenetic targeted therapy to reverse or halt the aging process due to an induced decline in stem cell maintenance and/ or function over time. Recent investigations have attempted to map the genome wide epigenetic changes associated with adult stem cells during aging, however with this area still in its infancy, we will endeavour to encapsulate the epigenetic hallmarks of aging stem cells by examining one of the most affected areas of the body and probably the most studied to date, the bone marrow and its associated resident stem cells, hematopoietic stem cells (HSC) and mesenchymal stem cells (MSC).

### Hematopoietic Stem cells

Aging has been most extensively studied in the hematopoietic system where it is synonymous to a change in lineage potential as there is a decline in lymphoid potential, and increase in myeloid potential, increased autoimmunity, elevated levels of ROS, accrual of DNA damage and enhanced prevalence of hematopoietic malignancies [53, 54]. Like many postnatal stem cells, HSC succumb to age-associated decline in function associated with specific gene expression changes. Gene expression analyses of young and old HSC have revealed that genes involved in inflammatory and stress responses are upregulated, whereas genes involved in DNA repair and chromatin silencing are generally downregulated [55]. These findings mirror observations in aged HSC, which exhibit increased DNA damage possibly due to deregulated chromatin remodelling genes leading to defective DNA repair [56]. Specifically, 1,337 genes were reported to be upregulated, whereas 1,297 were found to be down regulated during HSC aging. Gene ontology analysis revealed that genes upregulated included genes involved in cell adhesion, proliferation and ribosome biogenesis, whereas genes that were down regulated included genes involved in DNA base excision repair, DNA replication and the cell cycle. Gene set enrichment analyses has uncovered genes that were upregulated during aging consisted of HSC specific genes in agreement with the increased stem cell self-renewal [56].

In another recent study, HSC from aged and young mice were analysed for gene expression. Microarray analysis of c-kit positive HSC found 1,600 genes that were upregulated, and 1,500 genes downregulated during aging [55]. The genes found to be upregulated were genes involved in nitric oxide mediated signalling, protein folding and inflammatory responses. In agreement with other reports, genes that were downregulated were those involved in preserving genomic integrity such as chromatin remodelling and repair such as HDAC1,5 and 6 and Dnmt3b [55].

With technological advances in chromatin immunoprecipitation and whole genome sequencing (ChiPseq), the epigenome can now be interrogated using a low number of primitive rare cell types to map the epigenomic landscape. It has become increasingly apparent that epigenetic modifications are centrally important to the aging process, as epigenetic drift becomes a hallmark feature of aging cells [57]. In the hematopoietic system, aging is synonymous to a change in lineage potential as there is a decline in lymphoid potential, increase in myeloid cell numbers, increased autoimmunity, elevated levels of ROS, accrual of DNA damage and enhanced prevalence of hematopoietic malignancies [58-62]. Overall, transcriptome analyses of aging HSC show clear differences in gene expression patterns, suggesting crucial age associated changes occurring at the epigenetic level [58].

### DNA Methylation/Hydroxymethylation in HSC Aging

To date, the most extensively studied epigenetic modification is methylation of cytosine residues at the carbon 5 position - 5mc [63]. There are 28 million CpG sites in the human genome with many CpG dinucleotides existing outside gene promoters and CpG islands [64]. Examination of DNA methylation changes during aging have shown a general loss of methylation across the genome [63, 65, 66]. One of the most comprehensive studies examining causative mechanisms of whole blood aging examined large scale genome wide DNA methylation patterns in 738 individuals, using MBD-based capture and sequencing [67], which led to the discovery of 70 age related differentially regulated methylation regions (DMR). This consisted of 42 differentially DMR showing hypomethylation with age and 28 showing hypermethylation. Most of the changes were found to be in CpG islands or CpG shores, situated upstream from transcription start sites with enrichment towards transcription factor binding sites. None of the hypomethylated DNA regions were in CGI but were prevalent in DNase1 hypersensitive clusters, which represent regions of open chromatin. In contrast, hypermethylated regions were enriched in CGIs and displayed a greater preference for exons and upstream regions close to transcription start sites. Examination of the hypomethylated regions and cross referencing them to studies using pluripotent stem cells during senescence, found regions of hypomethylated DMRs enriched for the transcription repressor, CTCF, histone lysine methyltransferase EZH2 and H2AZ. Hypomethylated DMRs were also enriched for regions showing H3K27 acetylation, H3K4 methylation (di and tri), dimethylation and H3K9 acetylation. These marks are classical epigenetic signatures for active gene transcription, and is consistent with hypomethylated regions mapping to regions corresponding to DNase1 hypersensitive sites. These findings are in agreement with studies that link increased H3K4 trimethylation with longevity [31, 32]. Network analysis revealed that the hematopoietic transcription factors PBX3 (pre-B cell leukaemia homeobox 3), HOXB8 and MEIS1 are central to these regions. Similar to previous reports, these studies also found a high representation of protocadherins, HOXB family members such as MEIS1 and HOXA9,13, D11 and D13 [67].

A direct connection with DNA methylation and HSC aging is illustrated with the specific deletion of DNMT1, which leads to lineage skewing towards myelopoiesis and defective self-renewal [68, 69], and is characteristic of natural HSC aging. Further evidence of the role of DNA methylation during this process comes from studies showing that DNMT3A deletion results in a loss in differentiation potential of HSC after serial transplantation [70], and loss of both DNMT3A and DNMT3B leads to a more severe effect [71].

The DNA demethylases known as Ten Eleven Translocated 1, 2, 3 (Tet1, Tet2, Tet3) have also been associated with aging. Given DNA methylation is an epigenetic mark that is altered during aging it is not surprising to find changes in expression of the DNA demethylases. They are responsible for hydrolysing methylated DNA (5hmC), a process needed for the removal of DNA methylation [72-74]. Quantitative mass spectrometry has revealed a reduction in 5hmC levels during HSC aging in both mouse and human HSC [56], indicating a reduction in 5hmC is an epigenetic hallmark of aging HSC with Tet2 mutations a contributing factor. Tet2 deletion resulted in enhanced self-renewal with an increased primitive compartment of both stem and progenitor cells and enhanced myelopoiesis with declined lymphopoiesis [75, 76]. This implies that loss of Tet2 results in clonal expansion of HSC, skewed towards myelopoiesis and decreased lymphoid commitment, giving rise to the typical aging phenotype of HSC. It is also not surprising that mutations in Tet2 and DNMT’s seems to be initiating factors for the development of leukaemias [76, 77]. In agreement with these observations, aged HSC show differential expression of both DNMT and Tet enzymes, where mice deficient in any of these alleles show characteristics of aged HSC such as myeloid skewing and predisposition to cancer [76, 77]. In addition, the most frequently mutated genes in hematopoietic cells during aging are epigenetic regulators such as Tet2, DNMT3A and ASXL1 [78, 79]. Genome wide analysis of hydroxymethylation in total blood cells has found a significant (27.5%) and continuous reduction in 5hmC levels with age. Although sequencing the genomes of 100 aged individuals found somatic mutations in Tet2 and DNMT3A in 31 individuals, reduced levels of 5hmC were independent of the somatic mutations although reduced expression of epigenetic factors was not examined in these studies [80].

A distinguishing feature of HSC is that they are generally in a quiescent state to prolongue longevity [81-83]. At times of stress, the HSC undergo rapid proliferation, which is thought to be a contributing factor to global hypomethylation, a common feature observed in aged stem cell populations. Studies examining DNA methylation levels in pure populations of young and aged mouse HSC, have reported vast DNA methylation changes, that seem to be locus specific consisting of both hypo and hypermethylated DNA [84]. Interestingly, regions which display hypermethylation also overlap with PRC2 (polycomb repressor complex 2) genome rich regions and histone H3K27 methylation, known as classical repressor marks, suggesting a tight connection between these two marks to ensure gene repression [56, 59, 85]. What is often found in HSC is that changes in DNA methylation patterns during aging has little effect on gene expression, suggesting that DNA methylation may have heritable effects on the progeny arising from HSC. Studies examining DNA methylation have found that open chromatin regions that relate to lymphoid genes show increased DNA methylation, which during differentiation and aging results in decreased lymphoid and erythroid differentiation [84]. These are the two common lineages that are diminished during aging where DNA methylation changes seem to divert HSC to differentiate more towards the myeloid cell lineage. This raises the interesting possibility that altered DNA methylation in HSC during aging requires changes in histone modifications to effect gene expression patterns. Genome wide DNA methylation profiling in aging mice found that a quarter of the CpG sites showed age related changes in essentially all tissues examined with the most prominent changes for the most proliferative organs such as the gastrointestinal tract and blood system [86]. Changes in DNA methylation (hyper or hypo) occurs mainly on genes associated with lineage determination and PcG target genes are mainly hypermethylated [86]. Aging related hypomethylation is a well-known phenomenon for most tissues including leukocytes [87]. Whole genome bisulphite sequencing comparing HSC derived from young mice and old mice found that 70% of stem cell maintenance genes displayed hypomethylation during HSC aging, which correlated with their increased expression [55]. In the same study, hypermethylation was loosely associated with the binding sites of the transcription factor, PU.1, a key regulator of HSC differentiation [56]. These studies reinforce that changes in DNA methylation during aging enforce HSC renewal and inhibit differentiation. In addition, it has been found that ribosomal biogenesis genes are hypomethylated and are implicated as common targets during aging [56].

The epigenome is adaptive, capable of changing gene expression profiles based on external environmental influences. Factors such as diet and nutrient intake, inflammation and oxidative stress are capable of changing the methylation status of genes [88]. Although there is global hypomethylation during aging, various CpG islands in promoter regions are hypermethylated with increased age. Studies investigating peripheral blood samples during aging and obesity measured DNA methylation in conjunction with gene expression microarray analysis, encompassing 27,578 genomic sites in promoter regions (non promoter regions were not examined) [89]. The findings showed that most of the CpG were in CpG islands in the proximity of promoters. All together 125 probes showed changes in methylation, with 34 exhibiting reduced methylation and 91 displaying increased methylation. It is well known that restriction of calorie intake can increase lifespan, where restricted energy intake can induce elevated levels of DNMTs that increases the methylation of genes that would otherwise be upregulated during aging [90, 91]. Hence the different DNA methylation patterns observed in obese versus lean individuals could be related to increases in energy intake. The results in this study reveal mainly an increase in DNA methylation of CpG residues in proximity of genes during aging.

## Histone Modifications in HSC Aging

In addition to DNA methylation and hydroxymethylation, other studies have focused on changes in histone modifications during aging. In general, many lineage specific genes also demonstrate high levels of open chromatin structure, and H3K4me2, but have little expression in HSC meaning these genes are primed to be activated during differentiation. Genome wide studies have identified multiple age related genes regulated during aging, such as lysine specific demethylases (*kdm3a-b*, *kdm5-d* and *kdm6a-b*) with established roles in stem cell biology having decreased expression levels during aging [92-95]. One such demethylase, KDM5B, a H3K4 demethylase, normally highly expressed in primitive HSC, promotes differentiation by repressing genes involved in HSC self-renewal [96]. The age-related decrease in KDM5b may have a possible contribution to the loss of differentiation and expanded HSC compartment observed in aged mice and could possibly result in elevated levels of H3K4me2 and H3K4me3 in HSC.

Genome wide association of the key epigenetic marks of activation, H3K4me3, H3K36me3 and the epigenetic repressor H3K27me3 have been assessed in HSC during aging. Aged HSC show an age-related increase in H3K4me3, correlating with an increase in HSC gene expression [56]. This is in agreement with findings that aged HSC display a reduced expression of the H3K4me3 demethylase, KDM5b [96]. In contrast, changes in DNA methylation were found to have very little correlation with gene expression changes. This however could be related to the observation that DNA changes in HSC primes them for changes in gene expression at the differentiation stage. Whilst there were no changes in the levels of H3K27me3, significant changes in the distribution of H3K27me3 were observed as a substantial number of promoters showed an increase in H3K27me3. Further support for epigenetic modifiers in HSC aging is evident in the hematopoietic compartment, which is a reservoir for aged induced mutations that can lead to malignancies, where many of these mutations occur in epigenetic modifiers, including TET2, DNMT3 and EZH2 [97-100].

Similar studies comparing young and old HSC have found broader H3K4me3 peaks particularly in self-renewing associated genes, in agreement with DNA methylation studies, promoting gene transcription, expansion and differentiation of immature HSC [56]. While overall H3K4me3 levels remained comparable between young and old HSC, the peak intensities were observed to increase along TSS. This is in agreement with polycomb (PCG) regulators involved in H3K27me3 that have been shown to have significant roles in preventing HSC exhaustion [101, 102]. The PRC1 and PRC2 complexes contain H2AK119 ubiquitinase and H3K27 methyltransferase activity, respectively. Knockout of Bmi1 in mice, a component of the PRC1 complex, results in severe defects in HSC function and the derepression of the Ink4A locus which results in senescence of HSC [103-107]. Moreover, deletion of both Ink4A and p19Arf restores the defect in Bmi1 deficient HSC [105]. The same is evident when the PRC2 genes, Ezh1 and Eed are deleted in HSC, resulting in reduced self-renewal, however when Cdkn2A is also deleted, self-renewal is restored [106]. These results suggest that the PcG proteins safeguard against hematopoietic exhaustion by suppressing senescence and acting on the Ink4A/Arf19 locus. The converse occurs when *Bmi1*, *Ezh2*, *Eed*, *KDM2B* or *FBXL10* are overexpressed, leading to enhanced self-renewal and HSC potential during serial transplantation.

In addition to methylation, histone acetylation is also a key player in aging HSC. Loss of the deacetylase, Sirt1, in HSC leads to an increase in *HoxA9* expression and an increase in HSC numbers, under conditions of stress [108]. This ultimately leads to an increase in DNA damage and exhaustion of HSC in mice. In addition, Sirt3 is suppressed during aging and is essential for HSC under stress conditions. Overexpression of *Sirt3* leads to an enhanced regenerative capacity of HSC [109]. Furthermore, young HSC were found to have high levels of H4K16 acetylation, whereas a subset of old HSC exhibited a dramatic reduction in H4K16Ac. Interestingly, the distribution of H4K16Ac showed a more diffuse pattern in aged cells compared to young HSC.

During aging, hematopoietic cells exhibit a general hypomethylation with an overlap with DNase1 hypersensitive sites and open chromatin, which is also associated with H3K27 acetylation, H3K4me3 and H3K9 acetylation [56]. Hypermethylation is generally evident in CpG islands whereas hypomethylated DNA is not prevalent in these regions. In aged HSC, DNA hypomethylation is found on stem cell maintenance associated genes and genes involved in ribosome biogenesis, whereas hypermethylation is more closely associated with key differentiation genes such as PU.1, leading to skewed differentiation towards the myeloid lineage and decline in self-renewal capacity. The hypomethylated DNA signature is associated with active marks of histone methylation. H3K4me3 is associated with self-renewing and stemness genes and during aging there is an increase in H3K4me3 and a broader distribution around the TSS on self-renewing and stem cell genes [56]. This is consistent with studies that show a decrease in H3K4 demethylases such as KDM5B which expands the stem cell compartment and decreases differentiation [84].

Whilst it is known that DNA hypermethylation overlaps with H3K27me3 and repressive regions, studies have failed to find a significant difference in the levels of H3K27me3 during aging. However, H3K27me3 and polycomb group proteins appear to safeguard HSC from haemopoietic exhaustion by suppressing senescence and the *Ink4A* locus [110] Aging studies in HSC have consistently shown that changes in DNA methylation patterns during aging have little effect on gene expression, suggesting that DNA methylation may have heritable effects on the differentiated progeny arising from HSC. This raises the interesting possibility that altered DNA methylation in HSC during aging requires changes in histone modifications to effect gene expression patterns. In terms of 5hmC, more investigations are needed to examine changes in 5hmC in HSC during aging. Studies so far show that a reduction in 5hmC correlates with developmental skewing towards myelopoeisis at the expense of lymphopoiesis [75-77]. However, studies of aged HSC containing somatic mutations in Tet genes reported that other factors are also involved in the reduction in 5hmC.

## Mesenchymal Stem Cells and Aging

Mesenchymal stem/stromal cells (MSC) reside in the bone marrow and were originally identified as plastic adherent colony forming units (CFU-F) fibroblasts [111-113]. MSC have the capacity to self-renewal and give rise to multiple stromal lineages such as osteoblasts, adipocytes, chondrocytes, smooth muscle cells and myelosupportive stroma [114-116]. They exist as a heterogeneous population of stem/ progenitor cells with differential growth potentials, where the majority of CFU-F clonal populations undergo cell growth arrest before 20 population doublings, which is associated with a loss of telomerase activity during ex vivo expansion [115, 117-120]. With age, MSC numbers decline and their differentiation ability is skewed towards adipogenesis rather than osteogenesis[121-123] which is often a hallmark of diseases associated with an aging skeleton such as osteoporosis.

**Figure 2.**
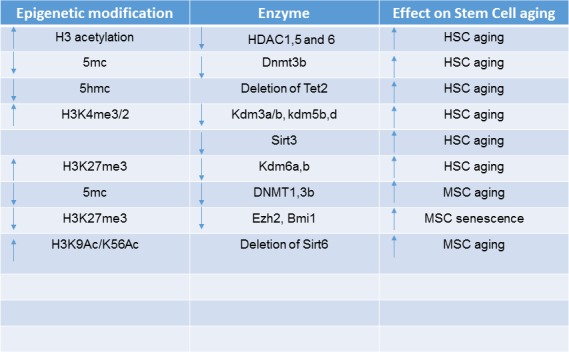
**Age Associated Epigenetic Changes in Aged HSC/MSC**. Histone and DNA modification marks in aged HSC and MSC are shown as well as their associated epigenetic enzymes. The effect on HSC/MSC aging is shown.

**Figure 3.**
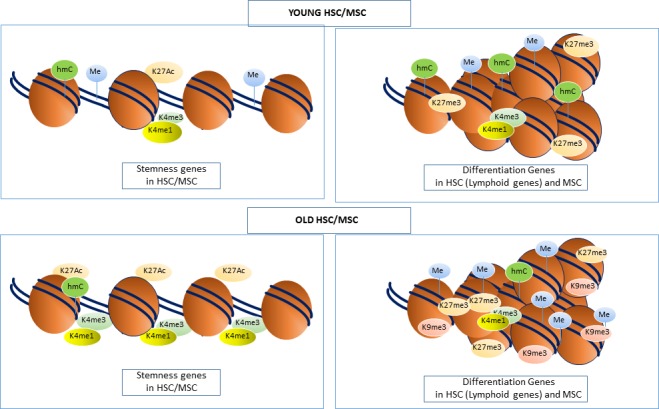
**Chromatin Structure of Stemness and differentiation genes in Aged HSC/MSC**. Histone and DNA modification marks in aged HSC and MSC consist of DNA methylation, H3K9me3 and H3K27me3 being more abundant along lineage/differentiation promoters therefore inhibiting transcription and differentiation. This is more apparent on lymphoid genes in HSC therefore skewing differentiation towards the myeloid lineage. Stemness genes contain an abundance of active marks such as H3K4me1, H3K4me3 and H3K27Ac leading to an open chromatin conformation and keeping the adult stem cells in an immature state.

Replicative cellular senescence is considered a form of in vitro aging sharing many of the molecular events associated with physiological aging. During ex vivo expansion, MSC undergo epigenetic and transcriptional changes, where the expression of osteogenic genes such as *collagen 1*, *alkaline phosphatase* (ALP), *Runx2*, *bone sialoprotein*, *osteocalcin* and *osteopontin* increase over continuous subculture, whereas the expression of stemness associated genes such as *Oct4*, *Nanog* and *Tert* decrease during subculture [124, 125]. These changes in gene expression profiles are associated with decreases in H3K9 and K14 acetylation for promoters of stemness genes and increases in acetylation for promoters of osteogenic genes. DNA methylation levels have also been found to change on the promoters and exons of these genes [125]. The decrease in expression of stemness genes is correlated to an observed decline in stem cell numbers in aged individuals, implying that decreases in histone acetylation may, in part, be a contributing mechanism to this. During senescence and aging, the expression of *DNMT1* and *DNMT3B* were found to be significantly downregulated in MSC [126], reflecting the global hypomethylation that is evident in aged MSC. Moreover, inhibition of DNMT1 and DNMT3B induced cellular senescence, supporting the potential role of these epigenetic modifiers in aging [126]. This was accompanied by hypomethylation of the *p16Ink4A* locus and *p21* promoter and a decrease in the levels of the PcG proteins, EZH2 and BMI1. The functions of EZH2 and BMI1 are not only integral to the regulation of MSC self-renewal and differentiation but also regulate expression of the p16Ink4A locus [127-132]. In agreement with these findings, we have found that transcription factors such as the helix loop helix transcription factor, Twist-1, promote the stemness of MSC and inhibit senescence, by promoting the recruitment of EZH2 to the *p16Ink4A* locus and inhibition of osteo/chondrogenic differentiation [128] [120, 133]. A summary of the epigenetic modifiers and modifications in HSC aging can be seen in Figure 2 and 3.

## DNA Methylation and Histone Modifications in MSC Aging

Two studies examining the DNA methylation profile of young and old MSC reported similar DNA methylation changes with MSC, following continuous cell passage until growth arrest in vitro and in MSC during in vivo aging [134, 135]. In the aging study, researchers used the human methylation bead ChIP array to characterise genome wide DNA methylation status of 429,789 CpG sites in primary human bone marrow derived MSC. The data showed that 64,142 autosomal CpG sites were differentially methylated between MSC of young and old individuals, of which 18,735 were hypermethylated and 45,407 were hypomethylated [135]. This is similar to what has been observed in HSC and other cell types, where aging is associated with a general decrease in DNA methylation. Both hyper and hypomethylated regions were represented in non-CGI and intragenic DNA regions, whereas hypermethylated regions were also present in CGIs. In the same study, the researchers compared the changes in CpG methylation to CpG methylation changes in aged glial, blood and neuronal cells, in order to identify deregulated methylated regions associated with aging rather than cell specific epigenetic differences. This approach identified a small but specific overlap for both hypo and hypermethylated DNA. When compared with databases mapping histone modifications in MSC, hypermethylated CpG sites in the common regions were found to be highly significant for the repressive histone marks H3K9me3, H3K27me3 and EZH2. Moreover, the hypomethylated marks occurred at regions occupied by the active histone marks H3K4me1 [135]. These results highlight that age related hypermethylation in MSC is associated with repressive histone changes that are also present in other aged somatic cell types, associated with a general systemic change in DNA methylation irrespective of the cell lineage. The same is evident for hypomethylation and active histone marks such as H3K4me1. Interestingly, H3K4me1 is associated with enhancers, which act as long-range regulators of gene transcription. Studies have examined the epigenome of multiple adult stem cells from young and old donors and compared them to cancer cells to identify a non-tissue specific aging signature that is similar in cancer cells. These studies showed that aging associated DNA hypermethylation in blood occurred at bivalent domains that were associated with key developmental genes, which have a significant overlap with cancer related genes [136]. Subsequent studies discovered a core DNA methylation signature of 589 CpGs that were related to age, the majority of which were targeted by PcG. These sites were independent of gender, tissue or disease state and were highly represented in MSC populations [137]. Supportive studies using the Human Methylation 27 Bead ChIP array examined 27,578 CpG sites from 13,500 annotated genes in young and old donors and identified 295 CpG sites that were hypermethylated and 349 sites that were hypomethylated [134]. Hypermethylated sites corresponded to genes such as *HoxA2*, *A5* and *A6*, *Runx2* and *DLX5*, known to be essential for osteogenic differentiation. Gene ontology classification showed that over represented hypermethylated genes were involved in limb morphogenesis, whereas hypomethylated genes were involved in sequence specific DNA binding. Given the overall decline in DNA methylation, subsequent studies have investigated whether reducing the levels of DNA methylation can potentiate the aging phenotype. Evidence for this can be seen in DNMT1 null mice which exhibit a decrease in overall bone mineral density with age, with associated increases in the incidence of lung and liver tumours, increased body fat content, and increased cognitive impairment [138].

Recently, the genomic distribution of 5hmC was studied in aged MSC. These analyses identified 134,693 hydroxymethylated CpG sites present in individuals ranging from the age of 2 to 89 years [139]. The 5hmC sites were mainly concentrated on introns and to a lower extent on promoters, 5’UTR and exons. Interestingly, there was a statistically significant association with the enhancer marker H3K4me1, but not H3K27Ac, illustrating its association with poised enhancers. Aged MSC showed 1,631 differentially hydroxymethylated CpG sites, in which 48% gained 5hmC and 52% lost 5hmC. Gene ontology analyses showed that both hyper and hypo hydroxymethylated DNA was enriched in genes specific for development. Specifically, hyperhydroxy-methylated were associated with morphogenesis whereas hypo-hydroxymethylated was associated with differentiation. In addition, hyperhydroxymethylated regions overlapped with genomic regions with hypomethylated DNA [139].

Aging models have consistently yielded important information regarding molecular changes associated with aging. MSC aging has been studied in patients with Werner syndrome who present with common symptomatic hallmarks of degenerated mesodermal tissues including osteoporosis, atherosclerosis and greying hair [140-142]. In a recent study it was hypothesized that this is due to accelerated exhaustion of the MSC pool. To test this, researchers differentiated ESC WRN^-/-^ (ESC from patients with Werner Syndrome) cells into MSC. The MSC derived from these mutant ES cells expressed all the MSC markers and could properly differentiate into osteoblasts, chondrocytes and adipocytes [143]. When WRN^-/-^ MSC were serially passaged they exhibited loss of replicative potential, increased number of senescence associated β-galactosidase positive cells and upregulation of cell cycle inhibitors, p16Ink4A and p21 Waf1. When injected into the muscle of NOD/SCID mice, the cells exhibited a severe reduction in their capacity to form ectopic mineralized bone tissue [143]. In agreement with increased senescence, the WRN deficient MSC also exhibited increased rates of DNA damage as shown by the appearance of p53BP1/yH2AX and phosphorylated ATM/ATR substrates and shorter telomere lengths. Examination of genome wide H3K27me3, K9me3, K4me3 and 5mc revealed no significant changes except for a significant decrease in H3K9me3. This is in agreement with the loss of heterochromatin associated with aging in all species examined thus far. There were 73 large genomic regions in total containing H3K9me3 in normal cells, where 38% of these were lost in MSC WRN^-/-^ cells [143]. The epigenetic modifications were found to reside predominantly in subtelomeric or sub centromeric regions. Furthermore, ChIP experiments showed that H3K9me3 localised to centromeric chromatin regions. In WRN depleted cells there was an increase in γH2Ax on centromeres and loss of H3K9me3. Immunprecipitation experiments revealed a complex between WRN, the H3K9 methyltransferase SUV39H1, HP1a and nuclear envelop proteins that bring HP1a to the complex. To test the importance of this complex, all components were individually knocked down, resulting in a significant reduction in H3K9me3 and overall induction of cellular senescence. In contrast, overexpression of these genes in WRN deficient cells caused increased H3K9me3 and maintained cell viability. In agreement with these findings, analysis of gene expression levels of *WRN*, *SUV39H1*, *HP1*, *LAP2b* and H3K9me3 levels between young and old dental pulp stem cells were associated with a general decline during age [143].

In another study examining histone associated mechanisms leading to cellular senescence, it was found that serial passaging of human MSC led to the systematic decrease in levels of the histone methyltransferase, *Ezh2*, which coincided with increases in the H3K27me3 demethylase, *JMJD3* and increased expression of *p16* and *p14* [128]. One critical factor that maintains the stemness of MSC is the βHLH transcription factor, Twist-1, which was found to prevent senescence of MSC by recruiting EZH2 to the *p16*/*p14* promoters inducing H3K27me3 and inhibiting their expression [128]. In a related study it was found that HDAC inhibitors induced senescence of MSC, which was associated with a decrease in the polycomb proteins, *Ezh2*, *Bmi1and SUZ12* and increased levels of *JMJD3* [144]. Moreover, this resulted in increased expression levels of *p16* corresponding to cellular senescence [144], supporting the central importance of *p16*/*p14*, studies on MSC senescence and aging, via DNA methylation repression of these genes. During replicative senescence, expression of *DNMT1* and *DNMT3B* are reduced, where reduction in DNA methylation results in upregulation of *p16* gene expression to drive cellular senescence [126].

A recent study verified the importance of histone deacetylation in MSC aging. It was recognised that MSC deficient in the histone deacetylase, Sirt6 displayed accelerated cellular senescence, dysregulated redox metabolism and increased sensitivity to oxidative stress [145].

To date, studies have reported that aged MSC, exhibit a global decrease in DNA methylation, which correlates with a reduced life span as they undergo senescence [134, 146]. Both hypermethylated and hypomethylated DNA regions are in non-CGI and intragenic DNA, whereas hypermethylated regions are also present in CGI [135]. In aged MSC, hypermethylated DNA overlaps with repressive chromatin marks including H3K9me3, H3K27me3 and EZH2, that correspond with genes involved in differentiation and limb morphogenesis including *HoxA2*, *A5*, *A6*, *Runx2* and *DLX5* [134]. Alternatively, hypomethylated regions overlap with the presence of H3K4me1, a marker for poised enhancers [135]. Other studies have found that 5hmC is also deregulated during aging with both hyper and hypohydroxymethylated regions of 5hmC was also found to be associated with the H3K4me1 mark, and genes corresponding to morphogenesis whereas hypohydroxymethylated regions correspond to differentiation genes which are also hyper methylated [139]. Histone modifications including H3K27me3/1, H3K4me3/1, H3K27Ac and H3K9me3/2 have not been studied in detail in aging MSC. Their deregulated genomic distribution and gene networks affected by their deregulation during aging is still an area of investigation that will reveal some fascinating insights into the mechanisms of aging related to the skeletal system. Collectively, these studies show a clear correlation of repressive DNA methylation marks and histone modifications clustering on differentiation genes during aging, inhibiting differentiation. A summary of the epigenetic modifiers and modifications in MSC aging can be seen in Figure 2 and 3.

## Future Perspectives of Epigenetic Research in Bone Marrow Stem Cell Aging

The importance of chromatin regulating aging and longevity is now well known however the discovery of epigenetic changes and associated epigenetic enzymes associated with aging is still in its infancy. Research in the age related epigenetic changes in HSC and MSC have revealed some common epigenetic changes. Increases in histone acetylation, decrease in DNA methylation and hydroxymethylation and genomic changes in H3K27me3 is evident. There is still an urgent need to obtain more detailed information on the epigenetic changes that drive the aging process with a focus on stem cells, quiescence and self- renewal to further define the epigenetic signature associated with stem cell aging. To add to this complexity, the role of miRNA and non-coding RNA in aging require further investigations to properly define the epigenetic influences affecting the aging of bone marrow stem cells. With more than 20 histone modifications and at least two DNA modifications currently known, mapping out all the epigenetic modifications and their complex changes during aging is essential to understand the epigenetics of aging. As epigenetic changes can be reversed, this area is a potential hot bed for exploitation in the area of aging as a strategy to epigenetically reprogram old stem cells into youthful functional stem cells. Knowledge in this field will also help bolster the self-renewal capabilities and ex vivo expansion of somatic stem cells to be used in stem cell based therapies as manipulation of their epigenome can increase the longevity and potency of cultured expanded stem cell preparations.
